# Autistic Adult Perspectives on Occupational Therapy for Autistic Children and Youth

**DOI:** 10.1177/15394492221103850

**Published:** 2022-06-17

**Authors:** Julia Sterman, Erin Gustafson, Lindsay Eisenmenger, Lizzie Hamm, Jules Edwards

**Affiliations:** 1 Edinburgh Napier University, UK; 2 University of Minnesota, Minneapolis, USA; 3 Minnesota Neurodivergent Education Advocacy and Therapy Services, White Bear Lake, USA; 4 Autistic, Typing, Minneapolis, MN, USA

**Keywords:** self-determination, evidence-based practice, occupational justice, autism

## Abstract

The Autistic community values neurodiversity-positive approaches rather than behavioral interventions for Autistic children; however, little is known about what that would look like in occupational therapy. Frequently, researchers seek parent perspectives for understanding Autistic children’s preferences, while to date insufficient attention has been paid to Autistic adults as valuable informants on the Autistic experience of Autistic children. The objective of the study was to understand Autistic adult perspectives on pediatric occupational therapy for Autistic children. We sought and thematically analyzed data from a large Facebook group and an occupational therapy podcast on Autistic values, needs, and experiences in pediatric occupational therapy. Participants described wanting therapy that supported Autistic identities rather than trying to “fix” children, changing environments or tasks to promote participation, and setting goals that address self-advocacy and autonomy. Occupational therapy practitioners should critically reflect on their practice’s alignment with Autistic values and start to shift their practice as needed.

## Introduction

Occupational therapy interventions and research for Autistic children and youth have predominantly focused on the needs of families from the perspectives of parents or other caregivers (Egilson et al., 2017; Wallisch et al., 2019). Parent perspectives provide invaluable understanding of their children’s daily lives; however, to develop authentic occupational therapy programming and support for Autistic children there is a growing acknowledgment of the need to include Autistic adult voices to provide insight into the lived experience of being Autistic (Laurent & Fede, 2021; Milton, 2014).

Occupational therapy interventions for Autistic children commonly include sensory strategies and Ayes Sensory Integration, task and environmental modification for difficult routines, behavioral approaches (e.g., Early Start Denver Model, Positive Behavioral Supports), animal-assisted therapies, and developing skills such as motor, social skills, and self-care skills (Kuhaneck, 2020).

Behavioral interventions such as promoting desired behaviors and reducing challenging behaviors are commonly used with Autistic children (O’Brien & Kuhaneck, 2020). Specific occupational therapy behavioral interventions may include pivotal response training, contingency methods, positive behavioral support, and applied behavioral analysis (ABA) (O’Brien & Kuhaneck, 2020). ABA and behavioral techniques from the field of ABA are emerging to be recognized as ineffective at best and abusive at worst (Sandoval-Norton & Shkedy, 2019), increasing incidence of post-traumatic stress disorder in people with previous ABA treatment (Kupferstein, 2018).

Social skills interventions are commonly used in occupational therapy with Autistic children to support them to engage with neurotypical children (Kuhaneck, 2020). However, this can promote “masking,” where Autistic individuals consciously or unconsciously change their behaviors to fit in with neurotypical society (Miller et al., 2021). Autistic adults describe that they learned to mask as young children and masking can increase stress and burnout, cause loss of identity, and lead to suicidal ideation (Miller et al., 2021). There is a growing understanding that, rather than having communication deficits, Autistic individuals express their empathy and communication differently than neurotypical individuals (Crompton, Ropar, et al., 2020; Kapp et al., 2013; Milton, 2012). Milton (2012), an Autistic researcher, coined the term, “double empathy problem” to explain how Autistic individuals’ perceived social skill deficits, arise from a lack of understanding and insight between Autistic and non-Autistic individuals. Autistic individuals are expected to follow neurotypical norms through masking; however, non-Autistic individuals rarely make the effort to better understand Autistic communication styles or change their communication style to match their Autistic conversation partners (Crompton, Sharp, et al., 2020; Milton, 2012). Communication is equally as effective with interactions between Autistic individuals as interactions between non-Autistic individuals (Crompton, Ropar, et al., 2020). Social interactions difficulties arise when the communication partners have varying neurotypes (Crompton, Sharp, et al., 2020). The solution to these communication difficulties is shifting the perception of autism and neurodivergence as a deficit to be fixed, to seeing it as a difference to be accommodated (Kapp et al., 2013).

Autistic individuals are clear that they would like neurodiversity-positive approaches rather than behavioral interventions (Laurent & Fede, 2021), and there is a nascent shift toward strengths-based paradigms in supporting Autistic children (Koenig, 2020). However, there is insufficient understanding of what neurodiversity-positive approaches should look like in occupational therapy. Change rhetoric tends to focus on not engaging in behavioral approaches with Autistic children, rather than what interventions occupational therapists should do with Autistic children. Most current literature lacks Autistic perspectives, including meaningful data representing what Autistic individuals want from their support services (Fletcher-Watson et al., 2019).

Although research has begun to incorporate the values of Autistic adults (Fletcher-Watson et al., 2019; Laurent & Fede, 2021), to date no research has leveraged Autistic adult perspectives to understand the potential occupational therapy needs and values of Autistic children and youth. The purpose of this study is to understand Autistic adult perspectives on occupational therapy for Autistic children and youth.

## Method

### Language and Reflexivity

This research is an effort to listen to and synthesize the values and desires of the Autistic community to support best practice for Autistic children and youth. The research team consisted of two neurotypical, allistic (non-Autistic), occupational therapy doctoral students; one neurodivergent, allistic, occupational therapy academic; one Autistic occupational therapist and parent to an Autistic child; and one Indigenous, Autistic advocate and writer who is a parent to Autistic children. The therapists on our research team acknowledge that they have previously provided services they would not do in the future or recommend to others, now that they are aware of the priorities and values of the Autistic community.

The Autistic community overwhelmingly prefers identity-affirming language (Bury et al., 2020), as autism is a central component of who they are. Thus, throughout this article, we will use “Autistic child” rather than child with autism (spectrum disorder). The capitalization of “Autistic” aligns with American Psychological Association (APA, 2020) guidelines for the Deaf and Blind communities and participant usage.

### Design

In this qualitative study, we sought to gather preexisting data that highlight the voices of Autistic adults on what they value in interventions for Autistic children and youth. Similar to the methods of Ryan and colleagues (2020) in using Facebook data, we purposively identified a Facebook group and podcast that could answer the research question. We gathered data from public pages of Autistic advocates on Facebook and one private Facebook group with approximately 70,000 members. This group is an Autistic-led space for parents, caregivers, and professionals seeking advice from Autistic adults about Autistic children or youth, and the largest of only a handful of such groups. We also sought pre-existing data from interview transcripts from Autistic guests on the *Two Sides of the Spectrum* podcast. This podcast is led by Meg Proctor, an occupational therapist who seeks to amplify Autistic voices and shift occupational therapy to be more strengths-based and in alignment with the neurodiversity movement through her organization’s continuing education offerings. To our knowledge, there are no other occupational therapy–focused podcasts amplifying Autistic voices. The internet as a communication modality aligns well with Autistic communication styles and is a space where Autistic individuals are often more comfortable than with face-to-face or phone conversations (Davidson, 2008). Social media platforms afford increased advocacy and connections for Autistic individuals (Davidson, 2008).

### Ethical Approval

Permission was granted from the founder of the Facebook group for data collection prior to applying for ethics approval. While this approval was sufficient to fulfill the requirements of the Ethics board, it is noted that Facebook presents a murky middle ground of being both private and public (Ditchfield & Meredith, 2018). While we only used posts that were shared within the large Facebook group, and did not read participant’s individual profiles, participants may not have understood the public nature of the words they were sharing in the group. Ethical approval was obtained from the University of Minnesota Institutional Review Board (STUDY00012546). Meg Proctor granted approval for use of the transcripts of her podcasts for analysis and dissemination. Data were anonymized by excluding any identifying information.

### Data Collection

Podcast data included 10 interview transcripts from the *Two Sides of the Spectrum* podcast, each approximately 40 to 60 min. Inclusion criteria of podcast transcripts included (a) the interview guest was Autistic and (b) the topic focused on interventions and services for children and youth. Table 1 describes the backgrounds of each podcast guest.

**Table 1.** Descriptions of Podcast Participants.

Name	Description
Sarah Selvaggi Hernandez	Autistic occupational therapist working to promote an identity-first approach to autism and occupational therapy. She is the founder of the Facebook page Autistic OT, which is a blog rooted in Autistic culture, occupational justice, and identity development.
Damian Milton	Autistic researcher and a part-time lecturer at the University of Kent. He consults with several organization and chairs the Participatory Autism Research Collective. The concept of the “Double Empathy Problem” comes from his research.
Lydia X. Z. Brown	Autistic attorney and disability justice advocate who focuses on violence against multiply marginalized disabled people. Lydia teaches at Georgetown University’s Disability Studies Program and founded the Fund for Community Reparations for Autistic People of Color’s Interdependence, Survival, and Empowerment.
Joy F. Johnson	Black, Autistic behavior specialist with a background in Applied Behavioral Analysis who runs a business called Spectrum Support where she partners with organizations, individuals, and families of Autistic people. Joy has master’s degrees in education and psychology and is currently a PhD candidate.
Jacquelyn Fede	Developmental psychologist, Autistic self-advocate, and co-founder of Autism Level Up! where they challenge occupational therapists to listen to Autistic voices and shift their ways of working with Autistic people.
Ido Kedar	Autistic-self advocate, writer, and a college student. He is a non-speaking Autistic person who uses a voice-output typing device to communicate. He has written a non-fiction book, “Ido in Autismland: Climbing out of Autism’s Silent Prison” as well as a fiction book, “In Two Worlds”.
Emily Lees	Autistic speech and language therapist working in specialist settings for Autistic children and teens in the UK. She is committed to adopting pro-neurodiversity approaches and seeks to be an advocate and role model for her students.
Elizabeth Sherman	Autistic occupational therapist and owner of Heart of Gold, Pittsburgh Therapy Project. She specializes in emotional-relational development, sensory integration, family-based occupational therapy support, and trauma-informed practice.
Sarah McCulloch	Autistic occupational therapist based in London and founder of the Autistic Empire, which an Autistic social organization built by and for Autistic adults to develop community-based resources on autism as a civic identity and provide practical tools and services for all Autistic people.
Greg Boheler	Autistic occupational therapy student and co-founder of the social media movement, OTs for Neurodiversity. He intends to leverage his divergent way of thinking and occupational therapy background as an advocate and a narrative shifter.

Facebook data consisted of 156 Facebook posts and comments from Autistic adults educating parents, carers, or professionals on supporting Autistic children. Facebook data inclusion criteria required that the post or comments be: (a) written by an Autistic member, (b) addressing an occupational therapy practice area, experience, and/or potential area of supports, and (c) focused on the needs of children and youth. The first three authors spent extended time reading posts and comments in the Facebook group to understand the types of questions asked and group norms prior to data collection (12 months for the first author, 4 months for the second and third authors). Data collection involved monitoring the group for relevant posts during the data collection period (May–June 2021), and using the within group search function to search for terms to elicit occupational therapy experiences such as: “occupational therapy,” “OT,” “services,” and “newly diagnosed,” and specific areas of practice such as “sensory,” “feeding,” “hair brushing,” and “social” from prior posts. We searched for Facebook pages by Autistic advocates (similar to blog postings) for potential inclusion; however, none met our inclusion criteria. Thus, we used advocacy pages solely as background information.

We intentionally sought Facebook and podcast data from Black, Indigenous, and other People of Color (BIPOC), due to the overrepresentation of White voices in Autism advocacy (Giwa Onaiwu, 2020). For example, we identified and considered for inclusion BIPOC Facebook pages, and intentionally included group posts that might elicit BIPOC perspectives such as hair care for Black Autistic children. However, we did not know the race or ethnicity of most Facebook participants given the forum’s potentially anonymous nature and did not intentionally collect participants’ perceived racial data.

### Data Analysis

The research team engaged in thematic data analysis due to its flexible approach to analyzing differing types of data (Braun & Clarke, 2006). Data from the podcast and Facebook group were imported into data management software (MAXQDA and Excel files). Data analysis and reduction began by two researchers inductively line-by-line coding podcast and Facebook data, while a third provided independent review of the codes and recommended modifications as needed. Table 2 provides example analysis steps. Analysis progressed with comparing and contrasting the two separate data sources and organizing the data based on shared concepts. Categories were developed and revised based on emerging understanding of the data. We used peer debriefing and consultation with Autistic authors to ensure the emerging themes resonated with Autistic experiences and to situate the research within the context of Autistic advocacy.

**Table 2.** Examples Within the Analytical Steps of Codes, Categories, and Themes.

Codes	Categories	Themes
• Need to change perception of autism • I will always be Autistic • OT does not fix, it should give support • Need perspective taking of the experience of autistic children • What’s the problem that needs fixing • Learn from Autistic people	• Listen to how autistic people talk about themselves/value themselves • Accept who Autistic people are	• Acceptance of Autistic children rather than trying to “fix” them
• Negative experiences with behavioral-focused therapies (including OT) • Behavior is communication • Areas for OTs to address • Activities of Daily Living • Fine motor/gross motor • Self-regulation • Sensory (including interoception)	• Therapy should support intrinsic motivation OR child-led, play-based therapy • Value in approaches to therapy (child, occupation, environment)	Changing environments or tasks rather than using behavioral strategies
• Shift from compliance-based services to promoting self-acceptance, autonomy, and advocacy • Values in outcomes/goals of therapy	• Support participation through • Changing expectations for the child • Changing tasks • Respecting and supporting independence, boundaries, and/or bodily autonomy • Supporting self-advocacy	Goals that address self-advocacy and autonomy

We maintained analysis trustworthiness in several ways. We ensured credibility through prolonged engagement in the Facebook group (field), data triangulation between podcast and Facebook group data, and investigator triangulation (Korstjens & Moser, 2018). An audit trail supported dependability and confirmability, and the researchers engaged in reflexivity through peer debriefing and ongoing journaling (Korstjens & Moser, 2018).

## Findings

### Acceptance of Autistic Children Rather Than Trying to “Fix” Them

Autistic adults often described society trying to change them by having them adhere to a neurotypical ideal. One Autistic podcast guest explained, “Please consider autism as a way of being . . . Autism is about the way that I think, and the way that I process, and the way that I interact with the world. It doesn’t come with negative or positive connotations” (Sarah S.). Many parents of Autistic children would post in the Facebook group asking what their newly diagnosed child needed for therapy and interventions, and the Autistic adults replying often responded with a similar statement, “just being Autistic does not require therapy” (FB). Instead, they would guide the poster to consider the child’s specific needs that require additional support by asking them “what do you think your child needs help with? . . .If he needs help communicating, try speech. If he needs help with motor skills, try occupational therapy or physical therapy” (FB).

The way the Autistic community perceived the value of occupational therapy was to support children’s specific needs, rather than assuming deficits and providing services simply based on their autism diagnosis. Many participants described how society trying to change their innate Autistic ways of acting and thinking negatively affected their mental well-being. One podcast participant provided a metaphor on society trying to change who she is because she is Autistic. “The problem with trying to force a round peg into a square hole isn’t that he doesn’t belong there, it’s that you’re breaking the peg trying” (Sarah S.). Many Facebook participants critiqued goals that are often presented in occupational therapy as trying to force Autistic children to be neurotypical, which was neither possible nor desirable:

Things like eye contact and ‘functional play’ are not appropriate goals for Autistic kids. These types of goals try to make kids indistinguishable from typical kids. What you want are goals that make your child into the happiest and healthiest Autistic kid they can be. (FB)

Thus, well-being and positive Autistic identity were valued over “masking” and being perceived by society as being neurotypical.

### Changing Environments or Tasks Rather Than Using Behavioral Strategies

Behavioral strategies are often focused on eliminating Autistic actions and thinking and replacing them with neurotypical actions and thinking. ABA was frequently recommended to parents of Autistic children, and the question of what is wrong with ABA was frequently asked by parents in the Facebook group. One Autistic participant replied,

Behavioral therapy is to make your child appear neurotypical. It teaches your child to mask and is compliance training. It is legalized abuse. Any occupational therapy or speech goals should never be to use mouth words or [to] verbalize anything. It should be on communication, whether it is AAC (Alternative and Augmentative Communication), helping to say certain letters or combinations, or sign language, but child-led and never forced. (FB)

Although occupational therapists are not ABA practitioners, participants described negative experiences with occupational therapy that included terms such as “compliance-based,” “withholding,” and “forcing eye contact,” and interventions that aimed for a child to tolerate sensations rather than supporting their sensory needs. Participants valued when occupational therapy sessions were child-led and used play and the child’s intrinsic motivation to address outcomes:

My 4-year-old son absolutely loves occupational therapy. There is no “work.” They go into a room with lots of crash mats and swings and play. He decides where he’d like to play, often with the swings or jumping/crashing first and then he moves to a quieter game, like a puzzle, drawing or a shopping game. (FB)

To increase participation, Autistic participants recommended modifying task demands and the environment rather than behavioral approaches. “If an Autistic child or adult is distressed and not complying, that’s indicating something is wrong with the environment or the demand, NOT the Autistic person” (FB). Participants frequently described a need to shift from focusing on changing a child’s behaviors, to understanding the reason for the behaviors and addressing the needs the behaviors illuminated. Behaviors often indicated tasks or environments that did not support the child’s needs. Changing environments shifts the onus for change away from Autistic children. “There’s so much attention on the Autistic person or the Autistic child and their development, when we should be looking at the systems and the people around them a lot more” (Damian).

We will use hair cutting as an illustrative example in this and the following theme. Hair cutting was a frequent Facebook group topic that required a shift in task expectations away from neurotypical or White standards. Recommendations from Autistic adults included: washing less, especially for Black hair; cutting hair less frequently or not at all; cutting hair completely off to not have to manage brushing and styling; and reducing demands on styling. A Facebook participant described her negative experience with hair care:

After years of having my own hair pulled and tamed into “pretty” styles causing blisters and hours of pain during and after styling I point blank refuse to do that unless my kid 100% wants it and asks on their own terms. I have completely dropped my hair expectations for me and them. (FB)

### Goals That Address Self-Advocacy and Autonomy

By accepting Autistic children for who they are and supporting changes to systems, tasks, and environments rather than people, occupational therapists can support Autistic autonomy. Participants desired outcomes for Autistic children to include the ability to self-advocate and have autonomy. Self-advocating to get needs and wants met includes receiving help from others. “Asking for help is a great skill to work on and put supports in place for. Supports need to be tailored to the individual, and what is within/just outside their comfort zone” (FB). Asking for help can support lifelong skills of self-advocacy and autonomy.

Returning to the hair cutting example, Facebook participants recommended giving children autonomy and choice throughout the hair cutting process from deciding what length and how to cut the hair, to the haircut itself. One Autistic parent described their haircutting routine with their Autistic child and ways they supported their child’s autonomy:

There’s no pressure, we stop every time my little one shows distress. My kid plays their tablet and is not expected to sit still. Sometimes the cut isn’t even but we don’t mind. We also have a comfort movie playing. Every movement is dictated by my child. Giving them total control over the situation builds trust. We tell my child everything that’s happening before, during, and after. My kid likes to see everything that will be used on their hair first. (FB)

Occupational therapists should consider ways that they can support skill development while also maintaining children’s bodily autonomy. One participant said, “Hand under hand I heard recently in an occupational therapy group and I thought that’s more respectful” (FB). Tasks facilitation can be modified to meet the desired objectives while also being respectful and promoting autonomy in Autistic clients.

Throughout their life, others often forced participants do things that were uncomfortable and violated their bodily autonomy, “I was taught to override my fear of social interaction, of unwarranted and inappropriate touch, and uncomfortable conversation all in the name of making me more socially acceptable to society” (FB). Autistic adults advocated against actions that violate Autistic children’s autonomy:

Share a bed with your cousin. Hold your sister’s hand. Take a picture with your grandpa. Most of these actions are harmless unless you have a child that has a natural aversion to forced social interaction. Every moment that an Autistic child is forced to interact socially in ways that are anxiety provoking and uncomfortable for them inadvertently teaches them to override their ability to choose and to override personal agency over their bodies. (FB)

Consequently, Autistic adults valued Autistic children to be empowered to make their own choices and express their wants and needs. An Autistic speech therapist valued “no” to support self-advocacy and autonomy for her Autistic clients. “Adults need to spend less time getting the kid to do what they want them to do and more time encouraging the times when the kid says, ‘No.’” (Joy). She valued respecting when a child says “no,” and always having “no” as an option, to respect their body and choices.

## Discussion

The purpose of this study is to understand Autistic adult perspectives on occupational therapy for Autistic children. Autistic adults describe wanting occupational therapists to: accept Autistic children’s Autistic identity, support interventions that change tasks and environments rather than focusing on attaining neurotypical skills, reject behavioral approaches, and create goals that address self-advocacy and autonomy for Autistic children to live an identity-affirming life. Participants in this study were clear that even if making someone less Autistic was possible, it would not be desirable, as autism is a lifelong neurotype that often requires supports and accommodations rather than a skill deficit. The themes identified within this research are interconnecting, accepting autism as a way of being and supporting Autistic identity leads to focus away from changing people to supporting self-advocacy and autonomy.

Using ecological models to analyze tasks and environments and make recommendations for change are core components of occupational therapy (Brown, 2019). Thus, the findings from the current research may be perceived as intuitive. However, many interventions for Autistic children continue to focus on skill development and remediating perceived deficits (O’Brien & Kuhaneck, 2020) rather than changing environments or tasks. Skill development has a place in occupational therapy intervention if it is individualized, aligns with the Autistic community’s values, and is developed in collaboration with an Autistic client (Chen & Patten, 2021). For example, many participants valued learning about how to accommodate sensory differences, including interoception. Autism in and of itself, does not need to be “treated.” Instead, occupational therapists can work with Autistic individuals to identify occupational performance challenges in their everyday lives, and determine changes or modifications to the environments and tasks (Chen & Patten, 2021). Focusing on the “why” of the task, rather than the task itself may support therapists, parents, and educators to let go of neurotypical standards and allow children to do tasks the way that works best for them (e.g., moving around the room to complete a writing task).

Autonomy and self-advocacy allow Autistic individuals to live the life that they want with appropriate supports. Behavioral interventions violate bodily autonomy, force compliance, and increase Autistic individuals’ risk for sexual and physical abuse (Sandoval-Norton & Shkedy, 2019). Due to challenges with understanding social cues for risk, Autistic children and adults are more likely to experience sexual assault than their neurotypical peers, and less likely to be able to communicate abuse they experience (Edelson, 2010). Consequently, self-advocacy and perception of bodily autonomy are important skills for their physical and emotional safety. If children’s bodily autonomy is not respected, they may not respect other’s bodily autonomy and boundaries. Whenever possible, consent should be sought from Autistic children, not simply from their parents. Children should get to decide what happens to their own bodies. Exceptions may include safety concerns or necessary medical treatment, but even in these situations, children should be given as much agency as possible. Children should be given opportunities to say “no,” indiscriminate of their communication modality. Thus, “no” should be programmed into all alternative and augmentative communication devices, and respected.

Autism is characterized by a lack of empathy and social awareness (Centers for Disease Control and Prevention, 2020), therefore, a common goal occupational therapists focus on with their Autistic and neurodivergent clients is “improving social skills.” However, building off research on double empathy and differing communication styles between and among neurotypes (Crompton, Ropar, et al., 2020; Milton, 2012), interest-based groups that support Autistic identity and communication among Autistic children may be more appropriate than groups that purport to teach neurotypical social interactions (Chen & Patten, 2021; Koenig, 2020) which can increase masking leading to burnout and suicidal ideation (Miller et al., 2021). Autistic children do not need to be taught neurotypical social skills. Instead, occupational therapists can support social engagement with Autistic children and neurotypical peers through advocacy and acceptance of differences in communication and socialization styles.

### Strength, Limitations, and Future Directions

To our knowledge, this is the first study to use Autistic adults to understand Autistic perspectives on occupational therapy services for Autistic children. Leveraging social media for data collection allowed conversations to happen in a natural environment, using the medium of text which may afford better Autistic communication. Facebook as a platform for research afforded the participation of international participants, including non-speaking or intermittently speaking Autistic adults, who may struggle with traditional interviews participation (Davidson, 2008).

The Facebook participants chose to spend time on a specific, large forum and may not represent the diversity of Autistic adults in terms of support needs, socio-economic background, and racial identity. It is important for future research to actively seek Autistic voices from differing backgrounds and with differing support needs.

To effectively collaborate with Autistic adults, occupational therapists should look toward groups that are consulting with Autistic adults for developing interventions for Autistic children (e.g., ASD Nest program (New York University, 2021) and that privilege Autistic voices throughout their programming (e.g., Autism Level Up (Laurent & Fede, 2021), and the Therapist Neurodiversity Collective (Therapist Neurodiversity Collective International, 2021). There is a large Autistic adult advocacy community that is trying to support the next generation of Autistic children. However, leveraging their voices requires occupational therapy practitioners to actively include Autistic adults in intervention development. A collaborative approach that values the voices of occupational therapists, families, Autistic adults, and other members of the multi-disciplinary team can support holistic client care.

Findings from this research are consistent values of the growing neurodiversity movement (Therapist Neurodiversity Collective International, 2021); however, these changes may be confronting to some occupational therapy practitioners. Similarly to how being anti-racist practitioners requires reflexivity prior to action (Sterman & Njelesani, 2021), providing neurodiversity affirming services should start with self-reflection on ways practitioners may be perpetuating ableist approaches. Occupational therapy is a caring profession, where practitioners want to support what is best for their clients. However, practitioners need to be open to change and learning. As Maya Angelou said, “Do the best you can until you know better. Then when you know better, do better.”

Autistic children will grow up to become Autistic adults. To identify which interventions and strategies best support Autistic children, future research must continue to synthesize Autistic adult views, needs, and desired occupational therapy outcomes as proxies for young, Autistic children who may not be able to communicate those concepts (Fletcher-Watson et al., 2019; Roche et al., 2021). This research study is a beginning in including Autistic voices in occupational therapy research for Autistic children and youth, but more must be done to build the evidence base. Until that time, occupational therapy practitioners can further their understanding of Autistic children’s needs by identifying the places where Autistic adults are advocating for their community through joining Autistic-led social media groups, listening to Autistic-led podcasts, and reading blogs, articles, and posts by Autistic authors. Until autism and neurodivergence are recognized as diversity, rather than a deficit among neurotypes, true meaningful and relevant outcomes within occupational therapy cannot be met.

## Conclusion

Autistic adults value accepting Autistic individuals as who they are rather than trying to change them to a neurotypical standard, modifying tasks and environments rather than compliance and behavioral approaches, and supporting goals that develop self-advocacy and autonomy. Autistic perspectives should inform all aspects of occupational therapy services for Autistic children.
